# Novel strategy for a high-yielding mAb-producing CHO strain (overexpression of non-coding RNA enhanced proliferation and improved mAb yield)

**DOI:** 10.1186/1753-6561-7-S6-O3

**Published:** 2013-12-04

**Authors:** Hisahiro Tabuchi

**Affiliations:** 1 Chugai Pharmaceutical Co., Ltd., 5-5-1 Ukima, Kitaku, Tokyo, Japan 115-8543

## Background

Innovation in mAb production is driven by strategies to increase yield. A host cell line constructed to overexpress TAUT (taurine transporter) produced a higher proportion of high-mAb-titer strains [1]. From these we selected a single TAUT/mAb strain that remained viable for as long as 1 month. Its improved viability is attributed to improved metabolic properties. It was also more productive (>100 pg/cell/day) and yielded more mAb (up to 8.1 g/L/31 days) than the parent cell line [2]. These results suggested that this host cell engineering strategy has great potential for the improvement of mAb-producing CHO cells.

## Results

**Figure 1:**
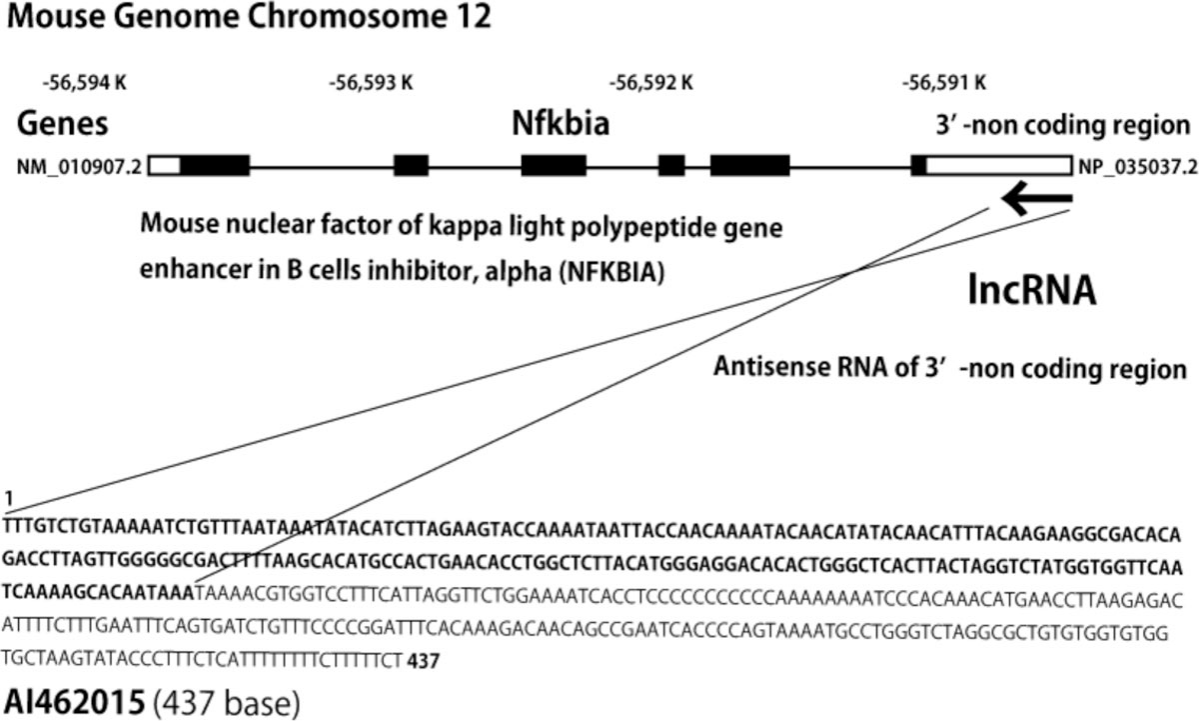
**The lncRNA is an antisense RNA of NFKBIA mRNA**.

Our present challenge was to achieve a high yield in a shorter culture period by modulating events in the nucleus by using non-coding RNA (ncRNA). We looked for long ncRNA (lncRNA) that was abnormally expressed in high-titer cells. A Mouse Genome 430 2.0 array (Affymetrix) identified the lncRNA (Figure 1) as a complementary sequence of the 3' non-coding region of mouse NFKBIA (NF-kappa-B inhibitor alpha) mRNA. NFKBIA is an important regulator of the transcription factor NFKB, a positive regulator of cell growth. Since NFKBIA suppresses NFKB function, inhibition of NFKBIA by overexpression of the lncRNA might further enhance cell proliferation. We genetically modified the TAUT/mAb strain to overexpress part of the lncRNA. The resulting co-overexpression strains gave increased yield, and one strain increased yield in a shorter culture period (up to 6.0 g/L/14 days from 3.9 g/L/14 days). Interestingly, however, this effect might not be due to enhancement of the NFKB-dependent promoter activity of the mAb expression plasmid because mAb production under EF-1α promoter without an NFKB binding site was also enhanced by overexpression of part of the lncRNA. Since overexpression of the partial sequence still functions as an antibody production enhancing sequence in mAb-producing cell lines, many unexpected functions from ncRNA-containing microRNA might exist.

## Conclusions

1. We found a lncRNA that was abnormally expressed in high-titer cells. It was identified as the antisense RNA of NFKBIA. Overexpression of part of the lncRNA suppressed NFKBIA mRNA.

2. Overexpression of part of the lncRNA improved CHO cell performance. The transporter/lncRNA co-overexpressing strain gave increased yield in a shorter culture period.

3. This effect might not be due to enhancement of the NFKB-dependent promoter of the mAb expression plasmid.
